# Risk Scores and Machine Learning to Identify Patients With Acute Periprosthetic Joints Infections That Will Likely Fail Classical Irrigation and Debridement

**DOI:** 10.3389/fmed.2021.550095

**Published:** 2021-05-03

**Authors:** Marjan Wouthuyzen-Bakker, Noam Shohat, Javad Parvizi, Alex Soriano

**Affiliations:** ^1^Department of Medical Microbiology and Infection Prevention, University Medical Center Groningen, University of Groningen, Groningen, Netherlands; ^2^Department of Orthopaedic Surgery, Yitzhak Shamir Medical Center, Zriffin, Israel; ^3^Department of Orthopaedic Surgery, Tel Aviv University, Tel Aviv, Israel; ^4^Department of Orthopaedic Surgery, Rothman Institute at Thomas Jefferson University Hospital, Philadelphia, PA, United States; ^5^Service of Infectious Diseases, Hospital Clínic, University of Barcelona, Barcelona, Spain

**Keywords:** debridement, implant retention, risk score, machine learning, failure, periprosthetic joint infection

## Abstract

The most preferred treatment for acute periprosthetic joint infection (PJI) is surgical debridement, antibiotics and retention of the implant (DAIR). The reported success of DAIR varies greatly and depends on a complex interplay of several host-related factors, duration of symptoms, the microorganism(s) causing the infection, its susceptibility to antibiotics and many others. Thus, there is a great clinical need to predict failure of the “classical” DAIR procedure so that this surgical option is offered to those most likely to succeed, but also to identify those patients who may benefit from more intensified antibiotic treatment regimens or new and innovative treatment strategies. In this review article, the current recommendations for DAIR will be discussed, a summary of independent risk factors for DAIR failure will be provided and the advantages and limitations of the clinical use of preoperative risk scores in early acute (post-surgical) and late acute (hematogenous) PJIs will be presented. In addition, the potential of implementing machine learning (artificial intelligence) in identifying patients who are at highest risk for failure of DAIR will be addressed. The ultimate goal is to maximally tailor and individualize treatment strategies and to avoid treatment generalization.

## Introduction

Success rates of the “classical” debridement, antibiotics, irrigation and implant retention (DAIR) for acute periprosthetic joint infections (PJI) vary widely, ranging from 30 to 90% (1–5). Apart from a thorough surgical debridement with exchange of modular components, many factors contribute to the success of DAIR; that includes shorter duration of symptoms, lack of patient comorbidities, a low bacterial inoculum and/or degree of inflammation at clinical presentation, a causative microorganism that is susceptible to antibiotics with anti-biofilm properties and many others (6–25). For this reason, being able to identify a category of patients who are likely to fail DAIR is essential, either to choose a different surgical procedure, to intensify antimicrobial treatment or to apply new innovative treatment strategies to increase the chance of treatment success. In this overview we will outline the current recommendations for DAIR treatment and discuss the limitations of these recommendations. In addition, we will address preoperative risk classification systems and the potential of machine learning to predict DAIR failure. These latter two show great potential to be used in clinical practice and may aid in clinical decision making.

## Who Should Receive DAIR According to the IDSA Guidelines

Since many different factors have been identified in literature as independent predictors for DAIR failure (Table 1) (6–25), it is a great challenge to select those patients who are the best candidates for DAIR. According to the IDSA guidelines published in 2013 (26), a DAIR is advised for patients with acute PJI, defined as a symptom duration of <3 weeks or, in case of early post-surgical infections, within 4 weeks of index arthroplasty. In addition, the prosthesis needs to be well-fixed, a sinus tract should be absent and the microorganism needs to be susceptible to oral antimicrobial agents with anti-biofilm activity. If these conditions are met, a DAIR is recommended, and in other situations revision of the implant is advised. Although this approach seems legitimate, it entails important limitations. First, it excludes a large subgroup of patients that may still benefit from DAIR. For example, in post-surgical cases it is advised to remove the infected implant when the index arthroplasty occurred more than 4 weeks ago. However, the process of mature biofilm formation varies substantially according to the type of causative microorganism and the inoculum size that contaminates the joint during surgery (27, 28). To therefore, exclude these patients as a candidate for a DAIR procedure is not justified. Indeed, Löwik et al. demonstrated an acceptable outcome of DAIR in patients presenting more than 4 weeks after the index arthroplasty as long as DAIR was performed within 4 week after the onset of symptoms and modular components were exchanged (29). In this category of patients, the prosthesis could still be retained in around 80% of patients without the need for life long suppressive antibiotic treatment. A second limitation of the IDSA recommendation concerning the indication for DAIR is the lack of distinction between early acute (post-surgical) and late acute (hematogenous) PJIs. This distinction may be critical, since several studies demonstrated a worse outcome in late acute PJIs treated with DAIR compared to early acute PJIs, in particular when caused by staphylococci (8, 13, 23, 30). Considering the difference in pathogenesis, and the chance of continuous seeding to the prosthetic joint in case of hematogenous infections (e.g., endocarditis), it is reasonable to assume that these infections should be approached differently as well. A third limitation of the IDSA guideline, is that the causative microorganism(s) and its susceptibility to antibiotics are often not known prior to surgery. A final limitation is the fact that implant- and host-related factors are not included in the decision-making model to determine appropriateness of DAIR. This may result in misclassifying a patient as a good candidate for DAIR while existing comorbidities may expose the patient to an increased risk for complications and failure. In addition, as the microorganism and its susceptibility to antibiotics is often not known prior to surgery, these implant- and host-related factors are of utmost importance to take into consideration.

**Table 1:** Summary of studies depicting independent predictors of DAIR failure in acute PJIs by using multivariate analysis.

**Reference**	**Author et al**.	**Year**	***N***	**Host, implant and surgical factors (known preoperatively)**	**aOR/aHR**	Microorganism and antibiotics (known postoperatively^*^)	**aOR/aHR**
(6)	Lora-Tamayo	2013	345	Immune suppressive drugs	2.23	Polymicrobial	1.77
				Serum CRP	1.22	Levofloxacin and rifampin^a^	0.42
				Exchange modular components	0.65	Vancomycin and rifampin^b^	0.29
				≥2 debridements	1.63	Bacteremia	1.81
(7)	Lora-Tamayo	2017	462	Rheumatoid arthritis	2.36		
				Revision prosthesis	1.37		
				Late post-surgical infection	2.20		
				Exchange modular components	0.60		
(8)	Wouthuyzen-Bakker	2018	340	Male sex	2.02	*S. aureus*	3.52
				Age > 80 years	2.60		
				COPD	2.90		
				Rheumatoid Arthritis	5.13		
				Fracture	5.39		
				Serum CRP > 150 mg/L	2.00		
				Exchange modular components	0.35		
(10)	Urish	2017	206	Symptoms > 7 days	1.68	*S. aureus*	0.59
(9)	Marculescu	2006	99	Sinus tract	2.84		
				Symptoms > 8 days	1.77		
(48)	Tornero	2016	143			Suboptimal antibiotic treatment^c^	4.92
(12)	Puhto	2015	113	Leukocytes > 10 × 10^9^/L	3.70	Ineffective empirical antibiotics	3.20
(13)	Vilchez	2011	65	Late acute PJI	2.57		
				≥2 debridements	4.61		
(14)	El Helou	2010	91			Rifampin in staphylococci PJI	0.11
(15)	Martínez-Pastor	2009	47	Serum CRP > 150 mg/L	3.57	No fluoroquinolone in Gram negative	9.09
(16)	Tornero	2015	222	Chronic renal failure	5.92		
				Liver cirrhosis	4.46		
				Femoral neck fracture	4.39		
				Revision prosthesis	4.34		
				Cemented prosthesis	8.71		
				Serum CRP > 115 mg/L	12.3		
(17)	Rodriguez-Pardo	2014	174	Chronic renal failure	2.56	Fluoroquinolone in Gram negative	0.23
(18)	Löwik	2018	386	Male sex	2.03		
				Left-sided prosthesis	1.80		
				Ischemic heart disease	1.84		
(19)	Tornero	2014	160	Liver cirrhosis	12.4	No fluoroquinolone in Gram negative	6.5
				Serum CRP > 120 mg/L	1.06		
(20)	Bergkvist	2016	35	Hip fracture	8.30		
(21)	Byren	2009	112	Revision prosthesis	3.10	*S. aureus*	2.9
				Arthroscopic procedure	4.20		
(22)	Vilchez	2011	53	Serum CRP > 220 mg/L	20.4		
				≥2 debridements	9.80		
(23)	Rodriguez	2010	50			*S. aureus*	5.3
(24)	Letouvet	2016	60	Number of prior surgeries	6.30	*S. aureus*	9.4
						Antibiotic treatment <3 months	20.0
(25)	Soriano	2006	47			*Enterococcus* spp. or MRSA	17.6

* *The presence of bacteremia, the causative microorganism and its susceptibility to antibiotics are sometimes known prior to DAIR, but in most cases not*.

a *Sub-group analysis of patients with a post-surgical PJI due to methicillin-susceptible S. aureus (MSSA)*.

b *Sub-group analysis of patients with a post-surgical PJI due to methicillin-resistant S. aureus (MRSA)*.

c *No rifampin for Gram positives and no fluoroquinolone for Gram negatives*.

*CRP, C-reactive protein; COPD, Chronic Obstructive Pulmonary Disease*.

## Preoperative Risk Scores to Predict DAIR Failure

To identify patients who are likely to fail DAIR, two preoperative risk scores have been proposed in literature; one for early acute (post-surgical) and one for late acute (hematogenous) PJIs (8, 16). These risk scores include only those variables that are known preoperatively without taking into account the causative microorganism and its susceptibility to antibiotics, mimicking the situation mostly encountered in clinical practice.

### KLIC-score for Early Acute (Post-Surgical) PJI

**Figure 1:**
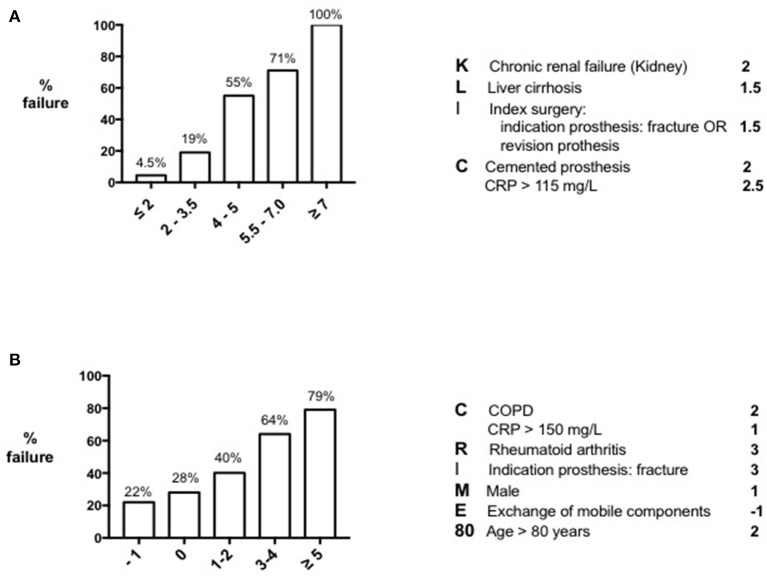
Preoperative risk scores for DAIR failure. KLIC-score for predicting DAIR failure in early acute (postsurgical) PJI **(A)** and CRIME80-score for predicting DAIR failure in late acute (hematogenous) PJI **(B)**.

In 2015, Tornero et al. published the KLIC-score as preoperative risk score for predicting DAIR failure in early acute PJI (16). The authors of this study examined a cohort of 222 patients who were within 3 months after the index surgery and who had no more than 3 weeks of symptoms prior to DAIR. DAIR failure was defined as the need for a second DAIR, implant removal, suppressive antibiotic treatment or infection-related death within 60 days after the initial irrigation and debridement. They analyzed in a univariate model several variables that were known preoperatively, like host-related factors, duration of symptoms, characteristics of the infected implant and serum inflammatory parameters, and developed a risk stratification score according to the beta-coefficients of the multivariate analysis (Figure 1A). Chronic **K**idney disease, **L**iver cirrhosis, the **I**ndex surgery (revision surgery or prosthesis indicated for a fracture), a **C**emented prosthesis and a **C**-reactive protein > 115 mg/l (**KLIC**) appeared to be the most prominent preoperative variables associated with failure. The score demonstrated 100% DAIR failure when having a preoperative score of more than six, and 4.5% when having a score lower than two. After this publication, three additional studies from other institutions validated the KLIC-score in their cohort of patients (18, 31, 32). All three institutions demonstrated the predictive power of the KLIC-score in patients with a very low or a very high score, but the score was less useful in patients with average scores. In addition, one study identified that other variables appeared to be more predictive in their cohort of patients compared to those defined in the KLIC (18), stressing the importance of differences in local epidemiology when implementing risk scores from an external cohort of patients.

### CRIME80-score for Late Acute (Hematogenous) PJI

Following the KLIC-score, Wouthuyzen-Bakker et al. performed the same statistical analysis in a large multicenter cohort of 340 patients with late acute PJIs (8). Late acute PJI was defined as the appearance of acute symptoms of infection occurring more than 3 months after the index arthroplasty, in a prior asymptomatic prosthetic joint. Patients with a sinus tract and/or patients with symptoms existing for longer than 3 weeks before DAIR were excluded. In contrast to the study of Tornero et al., a second DAIR procedure was not considered as failure, and failure could occur even 60-days after the initial debridement. In addition, the authors also included the exchange of mobile components as a valid preoperative variable, as the possibility to exchange it can be known prior to surgery as well. According to this analysis, **C**hronic obstructive pulmonary disease, a **C**-reactive protein > 150 mg/L, **R**heumatoid arthritis, fracture as **I**ndication for the prosthesis, **M**ale sex, not **E**xchanging the mobile components and an age > **80** years (**CRIME80**), were the strongest preoperative variables associated with failure (Figure 1B). The strength of prediction of the CRIME80-score was lower than the KLIC-score, starting with a baseline failure rate of 22%, and increasing to 79% with a score higher than four. It is important to note that the isolation of *Staphylococcus aureus* was one of the major predictors of failure in the late acute cohort. When *S. aureus* was the causative microorganism, the baseline failure rate was 43%, and the preoperative variables turned out to be less predictive in these cases. For this reason, the authors stress the importance of isolating the microorganism prior to deciding the surgical procedure. Unlike the KLIC-score, the CRIME80-score has not yet been validated in an external cohort of patients.

### Potential of Machine Learning (Artificial Intelligence) in Predicting DAIR Failure

Considering the complex interplay of factors associated with DAIR failure, regular statistical methods lack the finesse for more accurate and individualized predictions. The advantage of machine learning over regular statistical methods, like multivariate analysis, is its ability to actually learn from data input. Where multivariate analysis examines the correlation of variables and the strength of these correlations, machine learning learns from observations by using decision trees. The subsequently created algorithm is then able to process new input that has not been seen before. By this means, machine learning models are able to process more complex data, and by building precision models they are able to make more accurate predictions. Machine learning has become more and more popular in infection management (33). Recently, Shohat et al. used random forest analysis as a machine learning model to predict DAIR failure (34). The authors of this study analyzed more than 1,000 patients that underwent irrigation and debridement of a hip or knee prosthesis for acute PJI. The created algorithm had good discriminatory power, with an area under the curve of 0.74. Cross-validation, a model validation technique assessing the ability to process an independent dataset, showed similar probabilities, indicating a high accuracy of the model. Although the model still needs to be validated in an external cohort of patients, the created algorithm has great potential to be used in daily practice by easily entering patient data in a computer-based software or smartphone application, and may aid in clinical decision making and patient counseling. As the causative microorganism is of great influence on treatment outcome (7, 8), the authors of this study decided to include this variable in the analysis as well. Although its inclusion improves its predictive power, the microorganism needs to be entered to ensure the highest accuracy of the model, and thus, ideally should be known prior to surgery. The same holds for the presence or absence of bacteremia.

## To Tailor and Individualize Treatment Strategies

The described preoperative risk scores and machine learning model, can be applied in daily clinical practice, and may aid in the decision making process. When a patient has a high a priori risk for DAIR failure, immediate implant removal should be considered; not only to avoid surgery that is very likely to fail, but also to reduce the adverse effect of DAIR on subsequent surgical procedures (35, 36). Wouthuyzen-Bakker et al. analyzed the treatment outcome of immediate implant removal vs. DAIR in late acute PJIs by matching patients according to their preoperative CRIME80 score (37). The authors found that implant removal resulted in 83% treatment success in patients with a CRIME80 score ≥ 3, while the success was only 35% when treated with DAIR. No clear difference was observed between one- and two-stage exchange arthroplasties. Although a high CRIME80 score was logically associated with the presence of more comorbidities and old age, immediate implant removal was associated with a lower—instead of higher—mortality rate compared to DAIR. These data suggest that immediate implant removal is safe, even though the surgery in general is more aggressive.

A promising technique to potentially increase the success rate of DAIR, especially for difficult to treat microorganisms (e.g., multidrug resistant Gram negatives or rifampin resistant staphylococci), is to locally inject a selected cocktail of bacteriophages during surgery. Although future studies are needed to endorse this practice, its clinical success has been described as salvage therapy in relapsing *S. aureus* PJI (38). A main disadvantage though, is that the microorganism(s) causing the infection not only needs to be known prior to surgery, but the corresponding targeted bacteriophages need to be produced in the laboratory, before they can be applied. Since delaying DAIR increases the risk of treatment failure, the use of bacteriophage therapy for this indication is therefore, probably less feasible.

When a DAIR has been performed in a patient with a low preoperative risk score for failure, but the infection turns out to be caused by a microorganism that is resistant to biofilm active drugs, antibiotic duotherapy can be considered, particularly during the initial period. Adding fosfomycin to the antibiotic regimen as a second drug in infections with multidrug resistant Gram negatives or Gram positive microorganisms, or adding daptomycin for Gram positive infections, have shown great promise: both of the latter antibiotics have shown good antibiofilm properties *in vitro* and *in vivo* when used as part of a combination treatment (39–45). An alternative option is life-long antibiotic suppressive therapy, especially if patients are not eligible for additional surgery. According to a recent large multicenter cohort study with a follow-up period of 5 years, PJI can be controlled with antibiotic suppressive therapy in around 50% of cases (46). Another alternative strategy would be to apply new and more innovative treatments to control infection, like applying subcutaneous antibiotics for patients who do not tolerate oral antibiotics or for infections caused by multidrug resistant bacteria that lack an oral alternative. This proof of concept was demonstrated by Ferry et al. and was successful in 6 out of 10 patients (47). Considering the low chance of success for both treatment strategies, isolating the microorganism prior to surgery and choosing for implant removal in high risk patients would be preferable.

## Conclusion

Selecting those patients who are good candidates for a DAIR procedure is essential. Current IDSA recommendations for DAIR entail important limitations, and tools that also take into account other variables that are associated with DAIR failure are needed. Preoperative risk scores like the KLIC-score for early acute (post-surgical) and CRIME80-score for late acute (hematogenous) PJI could be helpful, especially when the microorganism is not known prior to surgery. In addition, machine learning shows great potential to predict failure more accurately compared to regular statistical methods. Implementing the aforementioned tools in daily care will help physicians tailor and individualize treatment strategies. Both described risk classification systems as well as the recently published machine learning model need clinical evaluation in larger external cohorts of patients to validate its predictive power.

## Author Contributions

All authors listed have made a substantial, direct and intellectual contribution to the work, and approved it for publication.

## Conflict of Interest

The authors declare that the research was conducted in the absence of any commercial or financial relationships that could be construed as a potential conflict of interest.
